# Activation of Sirtuin 3 and Maintenance of Mitochondrial Integrity by *N*-Acetylcysteine Protects Against Bisphenol A-Induced Kidney and Liver Toxicity in Rats

**DOI:** 10.3390/ijms20020267

**Published:** 2019-01-11

**Authors:** Wachirasek Peerapanyasut, Anongporn Kobroob, Siripong Palee, Nipon Chattipakorn, Orawan Wongmekiat

**Affiliations:** 1 Renal Physiology Unit, Department of Physiology, Faculty of Medicine, Chiang Mai University, Chiang Mai 50200, Thailand; wachirasek@hotmail.com; 2 Division of Physiology, School of Medical Sciences, University of Phayao, Phayao 56000, Thailand; anongpornkobroob@gmail.com; 3 Cardiac Electrophysiology Research and Training Center, Department of Physiology, Faculty of Medicine, Chiang Mai University, Chiang Mai 50200, Thailand; siripong.palee@gmail.com (S.P.); nchattip@gmail.com (N.C.)

**Keywords:** *N*-acetylcysteine, bisphenol A, toxicity, oxidative stress, mitochondria, sirtuin 3

## Abstract

Mitochondrial impairment ensuing from oxidative imbalance is related to adverse consequences of bisphenol A (BPA), a globally utilized industrial chemical. Recent evidence reveals sirtuin 3 (SIRT3) as a key regulator of mitochondrial homeostasis; however, its role in BPA toxicity remains unidentified. This study explored the potential benefits of *N*-acetylcysteine (NAC), an effective antioxidant, against BPA toxicity in the kidney and liver, and examined whether SIRT3 was involved in this condition. Male Wistar rats were fed with vehicle, BPA (5, 50 mg/kg), BPA (50 mg/kg) plus NAC (100 mg/kg) and were evaluated after 5 weeks. NAC treatment significantly diminished BPA-induced kidney and liver functional disorders, histopathological alterations, oxidative stress, and apoptosis. The increased mitochondrial reactive oxygen species, the disrupted membrane potential, the swelling, and the impaired mitochondrial fission caused by BPA were also mitigated upon concurrent treatment with NAC. The benefits of NAC were associated with enhanced AMPK-PGC-1α-SIRT3 signaling protein expressions, which led to decreased acetylation of superoxide dismutase 2 (SOD2) and increased expression of mitochondrial antioxidant manganese superoxide dismutase (MnSOD). The findings demonstrate the efficacy of NAC in protecting BPA-induced kidney and liver injury, which, in part, is mediated by activating SIRT3 and improving mitochondrial function, dynamics, and oxidative imbalance.

## 1. Introduction

Bisphenol A (BPA), one of the most utilized industrial chemicals worldwide, is commonly found in a variety of consumer products, particularly in those of polycarbonate plastics and epoxy resins [1]. Studies have reported that 90% of the general population has detectable levels of BPA [2] with occupationally exposed individuals having nearly 70 times higher BPA detected levels than in environmentally exposed populations [3] and, thus, BPA exposure is considered an inevitable situation. Importantly, several lines of evidence from epidemiological and experimental studies pointed towards the adverse health effects of BPA in multiple organ systems [4,5,6]. In addition, these unfavorable outcomes induced by BPA have been demonstrated to be associated with oxidative stress and disorders of the mitochondria [4,5].

Mitochondria have long been recognized as a major source of reactive oxygen (ROS) and nitrogen (RNS) species generation [7]. In recent years, the significant role of mitochondrial dynamics and biogenesis in the maintenance of mitochondrial homeostasis has yielded considerable interest. Accumulating data have shown alterations of these processes in many pathological settings initiated, particularly, by oxidative imbalance [7]. Therefore, maintenance of homeostasis within the mitochondria and prevention of redox imbalance may be a promising therapeutic strategy to cope with these disorders, including the toxicity caused by BPA. *N*-acetylcysteine (NAC), a glutathione precursor, is well accepted as a powerful antioxidant capable of protecting the cells and organs against oxidative damage in a number of abnormal circumstances [8,9]. The anti-inflammatory and anti-apoptotic effects of NAC have also been documented [9]. In the past few years, the mitochondrial protection by NAC has been demonstrated in several in vivo and in vitro study models [9,10,11], including BPA-induced neurotoxicity [12]. Even so, how NAC protects the mitochondria and the mechanisms responsible for its actions are as yet unknown.

The kidney and liver are two regulatory organs that contribute significantly to the maintenance of homeostasis within the body. Recent investigations have shown that nephrotoxicity as well as hepatotoxicity following BPA exposure is associated with mitochondrial oxidative stress and dysfunction [4,5]. However, the in-depth mechanisms responsible for mitochondrial changes and the signaling pathways leading to BPA-induced injuries remain to be clarified. The present study explored this issue with particular attention on the role of the silent information regulator T3 or sirtuin 3 (SIRT3) as this signaling molecule has recently been highlighted as playing an important role in the regulation of redox balance and mitochondrial homeostasis [13]. This study also tested the hypothesis that NAC has potential to combat the adverse renal and hepatic consequences of BPA through mitochondrial protection by activation of the SIRT3 signaling. If such is the case, the outcomes not only add novelty to a role for NAC as a mitochondrial SIRT3 activator, but give rise to a research challenge that may eventually translate into therapeutic benefits for patients with mitochondrial homeostatic disruptions.

## 2. Results

### 2.1. Effects of BPA Exposure and NAC Treatment on Body Weight, Kidney Weight, Liver Weight, Food and Water Intake

Oral BPA exposure for 5 consecutive weeks, either at 5 or 50 mg/kg and with or without NAC treatment, did not impact the rate of body weight gain including the intake of food and water (Table 1). Similarly, the relative weight of the kidney and liver in all studied groups was not affected by different treatments.

### 2.2. Effects of BPA Exposure and NAC Treatment on Kidney and Liver Functions

As shown in Table 2, the levels of blood urea nitrogen and serum creatinine in all the groups exposed to BPA did not alter from those in the control group. Creatinine clearance, urine protein-to-creatinine ratio and 24h urine protein excretion were also not affected by low dose of BPA exposure (5 mg/kg). However, increasing the dose of BPA to 50 mg/kg caused an increase in creatinine clearance, a marked rise in urine protein-to-creatinine ratio, including 24h proteinuria, compared to those of the low BPA-treated and the vehicle-treated groups (all *p* < 0.05). These alterations were counteracted when NAC was given along with the BPA (*p* < 0.05). Similar patterns were observed for the liver function test. Serum AST and ALT levels remained unchanged after exposure to low BPA but increased significantly following exposure to high BPA, and NAC treatment was able to minimize these changes, albeit to some degree (*p* < 0.05).

### 2.3. Effects of BPA Exposure and NAC Treatment on Histopathology of Kidney and Liver

**Figure 1:**
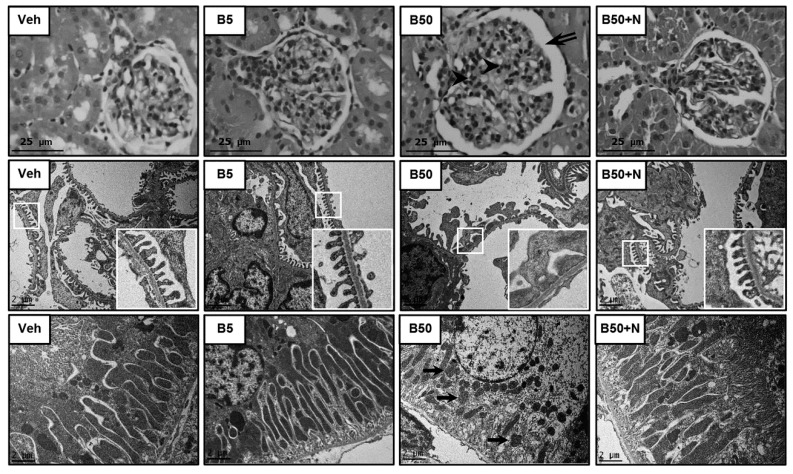
Photomicrographs of the kidney tissues following bisphenol A exposure and *N*-acetylcysteine treatment. Upper panel shows kidney sections stained with hematoxylin and eosin (H&E, 40×). Middle and lower panels show transmission electron micrographs of the glomerulus and renal tubules, respectively (original magnification: 3000×). The boxed areas are magnified in the right lower panel. Veh: vehicle-treated group; B5 and B50: bisphenol A-treated group at 5 and 50 mg/kg, respectively; B50 + N: BPA (50 mg/kg) plus *N*-acetylcysteine (100 mg/kg)-treated group. Double arrow, arrowhead, and arrow denote increased urinary space, mesangial proliferation, and mitochondrial swelling, respectively.

Light microscopic examinations of the kidney sections obtained from the B5-treated rats showed normal histological features with intact glomeruli and tubules similar to the vehicle-treated control rats (Figure 1, upper panel). Conversely, hilar hyperplasia, mesangial proliferation, and increased urinary space were evident in rats exposed to 50 mg/kg BPA for 5 consecutive weeks, which were mitigated with NAC administration. Consistent with light microscopy, electron photomicrographs from the B50-exposed rats revealed that the majority of podocytes were damaged, as detected by fusion and flattening of the podocyte foot processes (Figure 1, middle panel). Electron microscopy further demonstrated a decrease in mitochondrial number including an unusual mitochondrial ultrastructure such as swelling and loss of cristae in the proximal tubules of the B50-exposed group, whereas NAC treatment markedly attenuated these abnormalities (Figure 1, lower panel). However, no remarkable changes in glomerulus or tubule were observed on electron microscopic examination after exposure to BPA at 5 mg/kg for 5 weeks.

**Figure 2:**
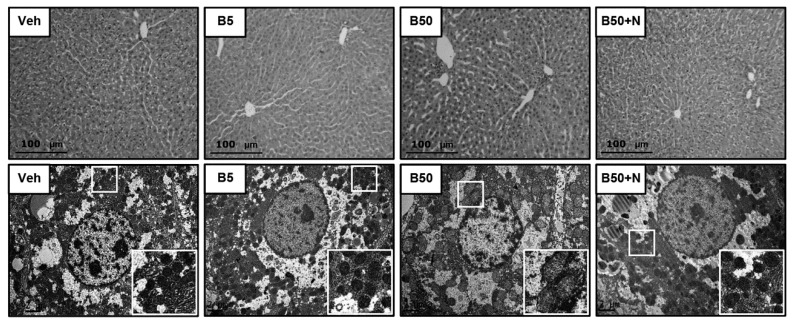
Photomicrographs of the liver tissues following bisphenol A exposure and *N*-acetylcysteine treatment. Upper panel shows liver sections stained with hematoxylin and eosin (H&E, 10×). Lower panel shows transmission electron micrographs of the hepatocyte (original magnification: 2000×). The boxed areas are magnified in the right lower panel. Veh: vehicle-treated group; B5 and B50: bisphenol A-treated group at 5 and 50 mg/kg, respectively; B50 + N: BPA (50 mg/kg) plus *N*-acetylcysteine (100 mg/kg)-treated group.

Hematoxylin and eosin stained liver tissues did not show any significant differences between the experimental groups (Figure 2, upper panel). At the electron microscopic level (Figure 2, lower panel), the vehicle-treated and the BPA 5 mg/kg-treated rats exhibited common fine structural features of hepatocytes, e.g., round nuclei with nucleoli, well-defined plasma as well as nuclear membrane, abundant mitochondria and intact mitochondrial cristae. However, a reduction of mitochondrial quantity in parallel with the occurrence of asymmetric mitochondrial swelling was evident in rats receiving BPA 50 mg/kg, and these conditions were obviously improved when NAC was delivered concurrently with BPA.

### 2.4. Effects of BPA Exposure and NAC Treatment on Kidney and Liver Oxidative Stress

**Figure 3:**
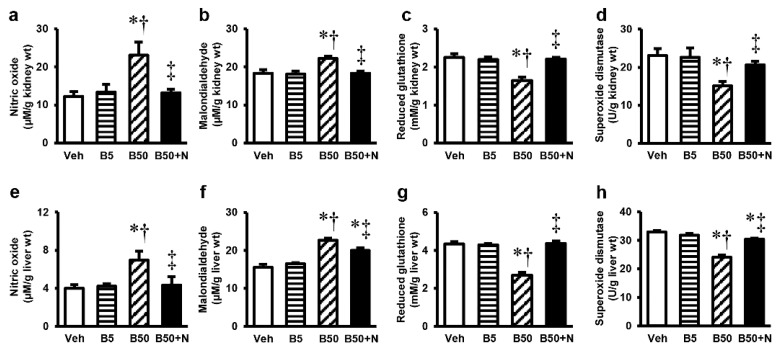
Effects of bisphenol A exposure and *N*-acetylcysteine treatment on kidney (**a**–**d**) and liver (**e**–**h**) oxidative stress. Values are means ± SEM (*n* = 6). Veh: vehicle-treated group; B5 and B50: bisphenol A-treated group at 5 and 50 mg/kg, respectively; B50 + N: BPA (50 mg/kg) plus *N*-acetylcysteine (100 mg/kg)-treated group. * *p* < 0.05 vs. Veh, ^†^ *p* < 0.05 vs. B5, ^‡^ *p* < 0.05 vs. B50.

Repeated exposure to BPA at 50 mg/kg for 5 weeks resulted in significantly increased NO and MDA but decreased GSH and SOD levels in the kidney tissues, whereas exposure at the dose of 5 mg/kg had no meaningful effects on these parameters. NAC treatment was capable of restoring all the changes in the kidney (Figure 3a–d) caused by this high dose of BPA. The consequences of BPA exposure and NAC treatment on liver oxidative stress (Figure 3e–h) were also found to follow the same pattern as that which occurred within the kidney. Although NAC therapy significantly reduced all oxidative changes caused by 50 mg/kg BPA, the MDA and SOD levels in the liver remained slightly different from the baseline values.

### 2.5. Effects of BPA Exposure and NAC Treatment on Kidney and Liver Mitochondrial Functions

**Figure 4:**
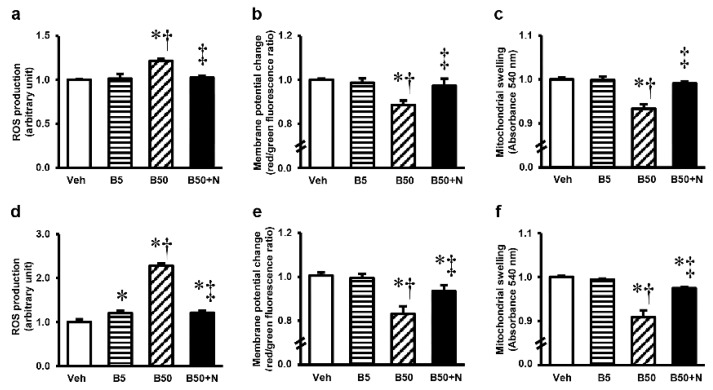
Effects of bisphenol A exposure and *N*-acetylcysteine treatment on kidney (**a**–**c**) and liver (**d**–**f**) mitochondrial functions. Values are means ± SEM (*n* = 6). Veh: vehicle-treated group; B5 and B50: bisphenol A-treated group at 5 and 50 mg/kg, respectively; B50 + N: BPA (50 mg/kg) plus *N*-acetylcysteine (100 mg/kg)-treated group. * *p* < 0.05 vs. Veh, ^†^ *p* < 0.05 vs. B5, ^‡^ *p* < 0.05 vs. B50.

The kidney mitochondrial function in rats receiving BPA 5 mg/kg remained unaltered compared to vehicle-receiving rats (Figure 4a–c). In contrast, a significant increase in mitochondrial ROS production (Figure 4a) and a decrease in mitochondrial membrane potential change (Figure 4b) along with mitochondrial swelling (Figure 4c) were evident after 5 weeks of BPA exposure at 50 mg/kg. NAC therapy prevented kidney mitochondrial dysfunction and brought all the changes caused by BPA back to normal. High BPA exposure, 50 mg/kg for 5 consecutive weeks, also induced liver mitochondrial disturbances that seemed to be more severe than those that appeared in the kidney mitochondria (Figure 4d–f). Though NAC was able to decrease the ROS production (Figure 4d), impede the dissipation of membrane potential (Figure 4e), and reduce the swelling (Figure 4f) of liver mitochondria substantially (*p* < 0.05), this modification did not completely recover.

### 2.6. Effects of BPA Exposure and NAC Treatment on the Levels of Pro-Caspase3, Cleaved-Caspase3, Pro-Apoptotic Bax and Anti-Apoptotic Bcl-2 in the Kidney and Liver

**Figure 5:**
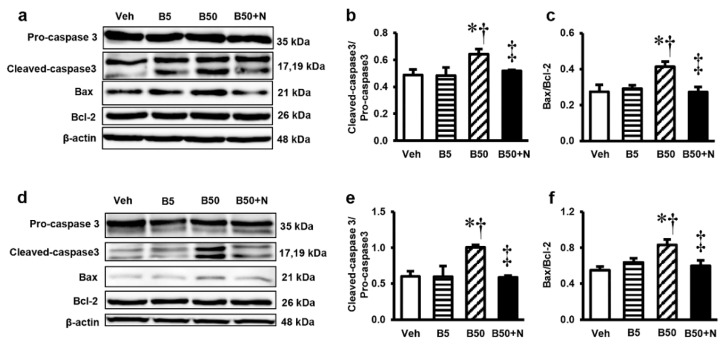
Representative images of western blots and the quantitative analyses of cleaved-caspase3/pro-caspase3 and Bax/Bcl-2 in renal cortical tissues (**a**–**c**) and liver tissues (**d**–**f**). Values are means ± SEM (*n* = 6). Veh: vehicle-treated group; B5 and B50: bisphenol A-treated group at 5 and 50 mg/kg, respectively; B50 + N: BPA (50 mg/kg) plus *N*-acetylcysteine (100 mg/kg)-treated group. * *p* < 0.05 vs. Veh, ^†^ *p* < 0.05 vs. B5, ^‡^ *p* < 0.05 vs. B50.

Analysis of protein expressions of pro-caspase3, cleaved-caspase3, pro-apoptotic Bax and anti-apoptotic Bcl-2 by western blotting were used to identify the apoptotic effect of BPA. Significant increases in the levels of cleaved-caspase3/pro-caspase3 including Bax/Bcl-2 ratio in the kidney (Figure 5a–c) and liver (Figure 5d–f) compared with those in the vehicle-treated controls were detected following exposure to BPA at 50 mg/kg, but not 5 mg/kg. In contrast, NAC supplementation to the BPA-exposed rats resulted in significant decreases in the levels of these apoptotic markers, observed in either the kidney or liver.

### 2.7. Effects of BPA Exposure and NAC Treatment on p-AMPK, AMPK, PGC-1α, SIRT3, Ac-SOD2 and SOD2 Expressions in the Kidney and Liver

**Figure 6:**
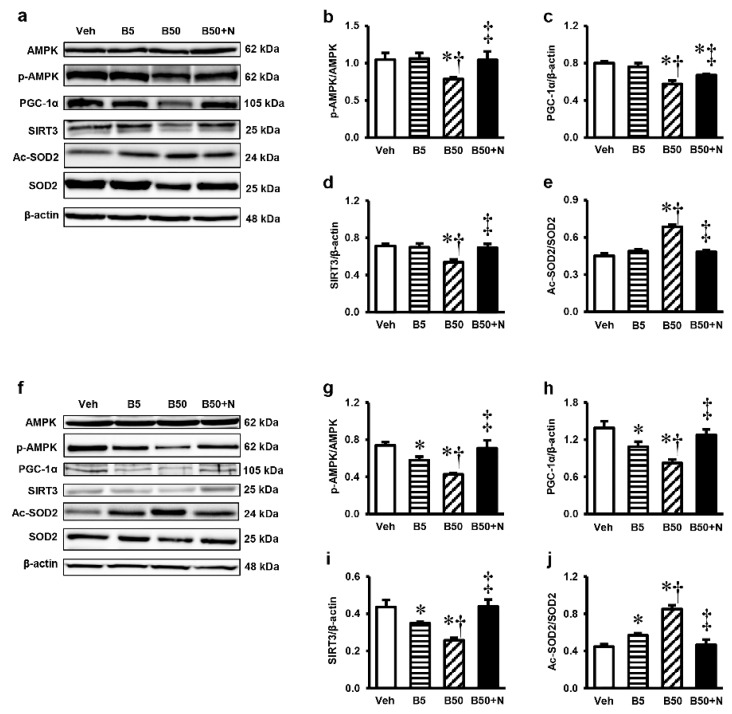
Representative images of western blots and the quantitative analyses of p-AMPK/AMPK, SIRT3/β-actin, PGC-1α/β-actin and Ac-SOD2/SOD2 in renal cortical tissues (**a**–**e**) and liver tissues (**f**–**j**). Values are means ± SEM (*n* = 6). Veh: vehicle-treated group; B5 and B50: bisphenol A-treated group at 5 and 50 mg/kg, respectively; B50 + N: BPA (50 mg/kg) plus *N*-acetylcysteine (100 mg/kg)-treated group. * *p* < 0.05 vs. Veh, ^†^ *p* < 0.05 vs. B5, ^‡^ *p* < 0.05 vs. B50.

To further investigate the signaling pathway involved in the effects of BPA and NAC, the p-AMPK, AMPK, PGC-1α, SIRT3, Ac-SOD2 and SOD2 protein expressions were evaluated in the renal cortex and liver. Western blot analyses showed that while BPA at 5 mg/kg did not affect any of these protein expressions, exposure to BPA at 50 mg/kg caused significant decreases in the renal levels of p-AMPK/AMPK ratio, PGC-1α, SIRT3, but increased the Ac-SOD2/SOD2 ratio (Figure 6a–e). These changes were significantly suppressed when NAC was given along with BPA. Regarding the liver (Figure 6f–j), significant reductions in the levels of p-AMPK/AMPK ratio, PGC-1α, SIRT3 while an increased Ac-SOD2/SOD2 ratio compared to the vehicle control were noticed in both BPA-treated groups. These effects appeared to occur in a dose-dependent manner and were abolished upon NAC therapy (all *p* < 0.05).

### 2.8. Effects of BPA Exposure and NAC Treatment on p-DRP, DRP1, and MFN2 in the Kidney and Liver

**Figure 7:**
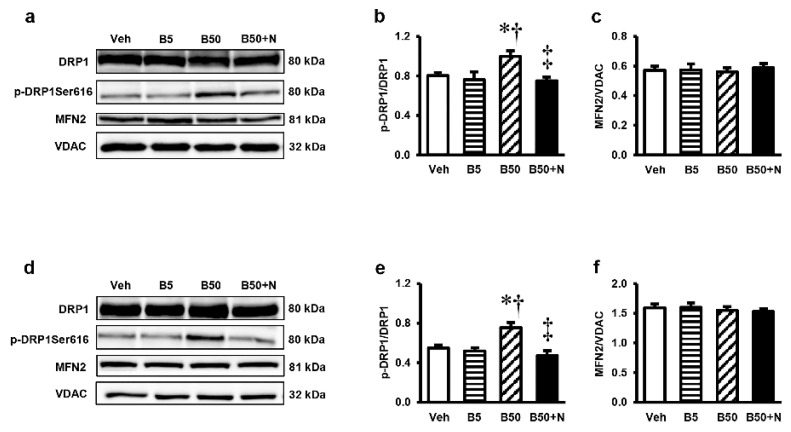
Representative images of western blots and the quantitative analyses of p-DRP1/DRP1 and MFN2/VDAC in renal cortical tissues (**a**–**c**) and liver tissues (**d**–**f**). Values are means ± SEM (*n* = 6). Veh: vehicle-treated group; B5 and B50: bisphenol A-treated group at 5 and 50 mg/kg, respectively; B50 + N: BPA (50 mg/kg) plus *N*-acetylcysteine (100 mg/kg)-treated group. * *p* < 0.05 vs. Veh, ^†^ *p* < 0.05 vs. B5, ^‡^ *p* < 0.05 vs. B50.

To determine whether BPA toxicity was associated with the alteration in mitochondrial dynamic, the proteins involved in mitochondrial fission and fusion were analyzed. There were significant increases in the levels of p-DRP1/DRP1 but no differences in the levels of MFN2/VDAC in the kidney (Figure 7a–c) and liver (Figure 7d–f) of the B50-treated group when compared with their corresponding vehicle group. Treatment with NAC was able to prevent the increases in p-DRP1/DRP1 caused by exposure of BPA at 50 mg/kg. However, at the dose of 5 mg/kg, there were no detectable changes in any of the mitochondrial dynamic markers in both the kidney and liver.

## 3. Discussion

The present study demonstrates for the first time the efficacy of NAC in combating the undesirable health hazard of BPA on the kidney and liver. The protective effects of NAC are mainly mediated by the maintenance of mitochondrial redox balance and the improvement of mitochondrial functional integrity. Another novelty emerging from this study is the compelling evidence that shows a link between the mitochondrial homeostatic role of SIRT3, BPA, and the therapeutic potential of NAC through the AMPK-PGC-1α-SIRT3-SOD2 axis.

BPA is of great interest because it is a high-volume industrial chemical used worldwide, with adverse health effects in multiple organ systems [4,5,6]. BPA toxicity has been extensively investigated in the context of environmental exposure, while the information regarding occupational exposure is scarce though the number of occupationally exposed individuals is increasing, particularly in the industrialized countries [14]. In BPA research, conflicting data have been reported, mostly due to a variety of dose and route of administration. As BPA exposure occurs mainly via ingestion, the present study has chosen to explore the effects of BPA via this route at two different concentrations. The dose of 5 mg/kg/day was selected to represent an environmental exposure as it is the NOAEL for BPA [15]. The amount of BPA used to mimic an occupational exposure was set at 50 mg/kg/day according to recent report that showed its profound effects in the rat kidney [5] and liver [16] after 5 consecutive weeks of exposure. In fact, it has been reported that occupationally exposed individuals had detectable BPA levels nearly 70 times higher than those in the general populations [3]. However, such high doses may not be appropriate as there was evidence shown an obvious increased incidence of acute toxicity with high mortality in rats after exposure to BPA close to 100 mg/kg/day [17].

In this study, oral exposure to BPA, either at 5 or 50 mg/kg/day, for 5 consecutive weeks did not affect the body weight, kidney and liver weights, including food and water intake, which is consistence with results obtained previously [5,16]. The functions of the kidney as well as liver remained intact after low-dose BPA exposure; however, alterations in both organs did occur upon exposure at the high concentration. Glomerular hyperfiltration (increased creatinine clearance) and proteinuria (increased urine protein-to-creatinine ratio as well as 24 h-urine protein excretion) were evident in rats exposed to BPA at 50 mg/kg, suggesting the development of glomerular hypertension and podocyte dysfunction. Further supportive evidence came from both light and electron microscopies showing various glomerular morphological changes such as dilated urinary space, hilar hyperplasia, mesangial proliferation and, most importantly, fusion and flattening of the podocyte foot processes. These findings are in line with recent publication showing BPA-mediated arterial hypertension and endothelial dysfunction by promoting oxidative and nitrosative stress via the activation of angiotensin II and calcium-calmodulin kinase II (CaMKII)-dependent uncoupling of endothelial nitric oxide synthase [18]. BPA has also been reported to induce podocytopathy with proteinuria by diminishing the synthesis of nephrin and podocin, the slit diaphragm proteins involved in the mechanisms of both proteinuria and podocyte survival [19]. Apart from effect of BPA at the glomerular level, our evidence from electron microscopy also displayed a reduction in mitochondrial number including an unusual mitochondrial ultrastructure such as swelling and loss of cristae in the renal proximal tubular cells of rats exposed to a high dose of BPA. As proteinuria is a consequence of two mechanisms, namely, an abnormal transglomerular passage of proteins due to increased permeability of glomerular capillary wall and an impaired reabsorption by the epithelial cells of the proximal tubules, it is possible that BPA-induced renal tubular damage may also be involved in the development of proteinuria in this study. Consistent with the kidney, our results indicated liver damage upon exposure to BPA 50 mg/kg for 5 consecutive weeks, as reflected by the elevated liver enzymes AST and ALT along with the detected hepatocyte ultrastructural changes under electron microscopy. The results obtained herein are in agreement with other previous studies regarding the hepatotoxicity induced by BPA [4,16,20].

Mitochondria have been accepted as a key target for BPA toxicity and the direct impact of BPA on the mitochondria has previously been reported [4,5]. Therefore, it is of interest to point out that BPA-provoked kidney and liver injuries in our study were instigated at the mitochondrial level through the induction of mitochondrial functional impairment, oxidative imbalance, apoptosis, and mitochondrial dynamic disturbances. This viewpoint was based on the present findings of mitochondrial dysfunction as evidenced by increased mitochondrial ROS production, decreased mitochondrial membrane potential, swelling of the mitochondria with abnormal mitochondrial morphology and quantities in the kidney and liver mitochondria isolated from the rats after 5 weeks of high BPA exposure. The decreases in expressions of mitochondrial manganese superoxide dismutase (MnSOD or SOD2), the activities of SOD, the levels of antioxidant GSH along with the increased levels of MDA and NO in the kidney and liver of BPA-exposed rats provided support of an alteration in mitochondrial redox homeostasis. Significant increases in the expression levels of Bax/Bcl-2 along with cleaved caspase-3/caspase-3 found after BPA exposure reflected the involvement of mitochondria-mediated apoptosis. Also, the increased mitochondrial fission protein expressions, p-DRP1/DRP1, in the kidney and liver after exposed to BPA are also evidence of mitochondrial dynamic disturbance.

SIRT3 is the primary mitochondrial NAD^+^-dependent protein deacetylase that plays a key role in maintaining mitochondrial vitality [21]. SIRT3 maintains redox homeostasis by regulating the function of electron transport chain complexes I and III and thereby prevents ROS generation. It also contributes to ROS detoxification by deacetylation of SOD2 and, as a result, activation of mitochondrial antioxidant enzyme SOD2 [22]. The role of SIRT3 as a new regulator of mitochondrial dynamics has recently been disclosed [22]. Interestingly, our findings obtained herein revealed that treatment with NAC concurrently with BPA was effective at protecting the kidney and liver mitochondria against functional and structural damages, oxidative imbalance, and restoring functional changes of the whole organ caused by BPA. Most importantly, our study is the first to demonstrate that the adverse effects of BPA are associated with reduced levels of SIRT3, and NAC counteracted these unfavorable consequences by restoration of SIRT3 expression. In accordance with our results, the importance of SIRT3 on mitochondrial-related organ injuries has recently been described in other models of acute kidney injury (AKI). It has been demonstrated that SIRT3 protects against AKI induced by ischemia-reperfusion [23], sepsis [24], and cisplatin [25], while SIRT3-deficient animals had more severe AKI and decreased survival rate. Compelling evidence from these studies also correlates the benefits of SIRT3 for the maintenance of mitochondrial function, dynamics, and redox homeostasis.

Significant additional findings in the present study are the correlation between the expressions of SIRT3, p-AMPK/AMPK ratio, PGC-1α and Ac-SOD2/SOD2 ratio after BPA exposure as well as upon NAC treatment. It was observed both in the kidney and liver that the reductions in SIRT3 expression after BPA exposure occurred simultaneously with the decreases in p-AMPK/AMPK and PGC-1α along with the increase in Ac-SOD2/SOD2 expressions. Treatment with NAC also exerted its protection against BPA toxicity through the reverse of the same signaling proteins. This is in line with the current knowledge revealing the AMPK-PGC-1α-SIRT3 as an essential signaling pathway for the regulation of mitochondrial oxidative stress as well as mitochondrial homeostasis [23]. Overall, our study demonstrates that activation of the AMPK-PGC-1α-SIRT3-SOD2 axis is, at least in part, a strategy for protection against the adverse effects of this important industrial chemical BPA, and further suggests that NAC is an effective substance for this purpose. However, further study using an SIRT3 knockout model to verify the effect of NAC as an SIRT3 activator is needed.

## 4. Materials and Methods

### 4.1. Drugs and Chemicals

Pentobarbital sodium (Nembutal^®^, Ceva Santé Animale, Libourne, France) was purchased from Ceva Animal Health Ltd. (Bangkok, Thailand). All other chemicals were obtained from Sigma Chemical Co. (St. Louis, MO, USA) and were of analytical grade.

### 4.2. Animals

Male Wistar rats (200–250 g) supplied by the National Laboratory Animal Center (Mahidol University, Salaya, Thailand) were maintained under a 12-h light/dark cycle at 24 ± 1 °C with food and water given ad libitum. All study protocols were approved by the Institutional Animal Care and Use Committee of the Faculty of Medicine, Chiang Mai University (Project number 02/2561 and 08/2561 approved on March 9 and 19, 2018, respectively) and conformed to the Guidelines for the Use of Laboratory Animals issued by the National Research Council of Thailand. A period of one-week acclimatization was allowed before starting the experiment.

### 4.3. Experimental Designs

Four groups of rats (*n* = 6 each) were studied. Group 1 (Veh) was used as control and the rats were fed with vehicle (corn oil). Group 2 (B5) and group 3 (B50) were given BPA orally at 5 and 50 mg/kg, respectively. Group 4 (B50 + N) received NAC (100 mg/kg) orally 60 min before BPA (50 mg/kg) administration. All treatments were given daily for 5 weeks. The low dose (5 mg/kg) of BPA was chosen as it has been shown to be the no observed adverse effect level (NOAEL) [15], while the high dose (50 mg/kg) was reported to induce oxidative stress in rat kidney [5] and liver [16]. The selected dose and regimen for NAC treatment were based on previous reports showing the potential to reverse cognitive dysfunction and oxidative stress induced by BPA in [12]. At the end of the study, rats were placed in metabolic cages for 24 h urine collections and blood samples were collected thereafter via abdominal aorta under pentobarbital sodium (60 mg/kg, i.p.) anesthesia. The kidneys and liver were quickly removed, and portions of the tissues from both organs were immediately taken for mitochondrial study or histopathological examinations. The remaining tissues were snap-frozen in liquid nitrogen and stored at −80 °C until biochemical analyses.

### 4.4. Determinations of Renal and Liver Functions

Serum samples were analyzed for urea nitrogen (BUN), creatinine, aspartate aminotransferase (AST), alanine aminotransferase (ALT), and urine samples were analyzed for protein and creatinine using AU480 chemistry analyzer (Beckman Coulter, Inc., Brea, CA, USA). The urine protein-to-creatinine ratio (UPCR) was computed directly, while creatinine clearance was estimated from the ratio of creatinine in urine/serum and the volume of urine produced and used as an index of glomerular filtration rate (GFR).

### 4.5. Determinations of Renal and Liver Oxidative Stress

Oxidative stress was assessed by detecting the levels of nitric oxide (NO), malondialdehyde (MDA), reduced glutathione (GSH), and superoxide dismutase (SOD) activity. Briefly, the kidney and liver tissues were homogenized in appropriate buffers using a Potter Elvehjem homogenizer (Wheaton Science, Millville, NJ, USA) and centrifuged at 10,000× *g* for 15 min at 4 °C. The obtained supernatants were assayed for all oxidative stress markers using commercial kits (Bioassay Systems, Hayward, CA, USA) according to the manufacturer’s instructions.

### 4.6. Histopathological Studies

Kidney and liver tissues were fixed in 10% neutral buffered formaldehyde, routinely processed according to standard histochemical methods, and finally embedded in paraffin. Sections were cut at 4 μm, stained with hematoxylin and eosin (H&E), and examined under a Leica DM750 photomicroscope (Leica Microsystems, Heerbrugg, Switzerland) by a pathologist blinded to the treatment.

### 4.7. Electron Microscopic Studies

The electron microscopic examination was performed following the procedure described by Peerapanyasut et al. [26] with slight modification. Briefly, pieces of renal cortical and liver tissues were fixed overnight with 2.5% glutaraldehyde in 0.1 M phosphate buffer (pH 7.4, 4 °C), postfixed in 2% phosphate-buffered osmium tetroxide, dehydrated in graded ethanol, and embedded in Epon resin using EMbed-812 embedding kit (Electron Microscopic Sciences, PA, USA). Sections (60–80 nm) were mounted on copper grids, stained with uranyl acetate and lead citrate, and examined using a JEM-2200 FS transmission electron microscope (JEOL, Tokyo, Japan).

### 4.8. Preparation of Mitochondrial Fractions and Mitochondrial Proteins

Kidney and liver mitochondria were isolated by differential centrifugation according to methods described by Kobroob et al. [27] and Sayeed et al. [28], respectively. Briefly, the tissues were homogenized in iced-cold isolation buffer (230 mM mannitol, 70 mM sucrose, 1 mM EDTA, and 10 mM Tris-HCl, pH 7.4) and, after centrifugation, the obtained mitochondrial pellets were resuspended in respiration buffer (250 mM sucrose, 5 mM KH_2_PO_4_, 10 mM Tris-HCl, 2 mg/mL BSA, pH 7.2, 4 °C). A bicinchoninic acid (BCA) assay was used to quantify the total protein content in the mitochondrial fraction as described previously [29].

### 4.9. Determination of Mitochondrial ROS Production

Mitochondrial ROS production was evaluated by the cell-permeable probe 2′,7′-dichlorofluorescin diacetate (DCFDA) using a protocol previously reported [27]. Mitochondria were stained with 2 µM DCFDA at 25 °C for 60 min. In the presence of ROS, a non-fluorescent DCFDA reacts with ROS and turns to a highly fluorescent dichlorofluorescin (DCF). The fluorescent intensity of DCF was quantified by a fluorescene microplate reader (Synergy^TM^ H4, BIOTEK^®^ Instruments, Inc., Vermont, USA) using excitation/emission of 485/530 nm. The magnitudes of ROS production were expressed as arbitrary units of fluorescence intensity of DCF.

### 4.10. Determination of Mitochondrial Membrane Potential (ΔΨm)

Mitochondrial membrane potential was determined using a fluorescent, lipophilic, cationic JC-1 dye (5,5′,6,6′-tetrachloro-1,1′,3,3′-tetraethylbenzimi-dazocarbocyanine iodide). JC-1 forms red-fluorescent aggregates at low membrane potentials as in polarized mitochondria, while it predominantly exists as green-fluorescent monomer at higher potentials. Mitochondria were incubated with JC-1 for 30 min at 37 °C and a fluorescene microplate reader (Synergy^TM^ H4, BIOTEK^®^ Instruments, Inc., Vermont, USA) operated at excitation/emission 535/590 and 485/530 nm was used to measure fluorescence intensity of the red-aggregate and green-monomer forms, respectively. The mitochondrial membrane potential was presented as the ratio of red/green fluorescence intensity, where a decrease in the ratio indicates mitochondrial depolarization and loss of membrane integrity [27].

### 4.11. Determination of Mitochondrial Swelling

Mitochondrial swelling was detected by a decrease in the mitochondrial absorbance according to the light-scattering method [30]. Briefly, mitochondrial suspension was monitored at 540 nm, 25 °C, every 1 min for 15 min in mitochondrial respiration buffer using a microplate reader (Synergy^TM^ H4, BIOTEK^®^ Instruments, Inc., Vermont, USA).

### 4.12. Western Blot Analysis

Renal cortical tissue and liver tissue were extracted in lysis buffer containing 20 mM Tris pH 6.8, 1 mM sodium orthovanadate, 5 mM sodium fluoride, and 1% protease inhibitor. Total protein in the lysates was determined using a Bio-Rad protein assay kit (Bio-Rad Laboratories, Hercules, CA, USA). Protein extracts were mixed with loading buffer (5% betamercaptoethanol, 0.05% bromophenol blue, 75 mM Tris pH 6.8, 2% SDS and 10% glycerol) and boiled at 95°C for 10 min. Protein samples were separated by electrophoresis on 10% SDS-PAGE and transferred to nitrocellulose membranes, which were blocked for 1 h with 5% bovine serum albumin in Tris-buffered saline-Tween 20 (TBST). The membranes were then incubated overnight at 4°C with primary antibodies against Ac-SOD2, Bax (Abcam, Cambridge, MA, USA), p-AMPK^Thr172^, PGC-1α (Millipore Corporation, USA), Bcl-2, caspase3, cleaved caspase3, AMPK, SIRT3, DRP1, p-DRP1^Ser616^, MFN2, SOD2, VDAC (Cell Signaling Technology, Danvers, MA, USA) and a loading control β-actin (Santa Cruz Biotechnology, Santa Cruz, CS, USA), followed by a horseradish peroxidase-conjugated secondary antibody at room temperature for 1 h. The blots were then visualized with an enhanced chemiluminescence prime western blotting (ECL) detection reagent and exposed using ChemiDoc™ Touch Imaging System (Life science AP, Bio-Rad, CA, USA). A densitometric analysis was performed using Image J program (National Institute of Health, Bethesda, MD, USA).

### 4.13. Statistical Analysis

Results are expressed as means ± SEM and were analyzed using SPSS 24.0 (SPSS Inc., Chicago, IL). One way analysis of variance (ANOVA) followed by Fisher’s LSD post hoc test was applied to determine the statistical differences among the groups. *p* < 0.05 was considered statistically significant.

## 5. Conclusions

The present results provide novel evidence to indicate the nephroprotective as well as hepatoprotective effects of NAC against BPA toxicity. The maintenance of redox balance, the preservation of dynamic equilibrium, and the improvement of functional integrity of the mitochondria are the underlying mechanisms responsible for the protection by NAC. These beneficial outcomes are, in part, mediated through the enhancement of AMPK-PGC-1α-SIRT3-SOD2 signaling pathway. The findings obtained open up a new perspective on the possible role of NAC as an SIRT3 activator, which may be useful in the prevention and treatment of other diseases involving mitochondrial impairment.

## Figures and Tables

**Table 1:** Effects of bisphenol A exposure and *N*-acetylcysteine treatment on body weight, kidney weight, liver weight, food and water intake.

Parameters	Veh	B5	B50	B50 + N
Initial BW (g)	240.00 ± 2.89	238.33 ± 2.79	239.17 ± 2.39	241.67 ± 1.05
BW gain (%)	60.80 ± 1.40	61.70 ± 1.56	59.62 ± 2.43	59.47 ± 1.41
KW/BW (*100)	0.59 ± 0.01	0.58 ± 0.01	0.59 ± 0.02	0.58 ± 0.01
LW/BW (*100)	4.12 ± 0.13	3.97 ± 0.08	4.15 ± 0.21	3.98 ± 0.15
Food intake (g/day)	26.56 ± 0.65	27.44 ± 0.58	27.22 ± 0.78	26.72 ± 0.50
Water intake (mL/day)	39.44 ± 1.27	38.89 ± 0.56	38.06 ± 1.32	38.61 ± 1.69

Values are means ± SEM (*n* = 6). Veh: vehicle-treated group; B5 and B50: bisphenol A-treated group at 5 and 50 mg/kg, respectively; B50 + N: BPA (50 mg/kg) plus *N*-acetylcysteine (100 mg/kg)-treated group; BW: body weight; KW: kidney weight; LW: liver weight.

**Table 2:** Effects of bisphenol A exposure and *N*-acetylcysteine treatment on kidney and liver functions.

Parameters	Veh	B5	B50	B50 + N
BUN (mg/dL)	21.85 ± 0.72	21.40 ± 0.50	21.35 ± 0.47	22.15 ± 1.08
SCr (mg/dL)	0.29 ± 0.01	0.30 ± 0.01	0.30 ± 0.01	0.28 ± 0.01
CCr (ml/min/g kidney wt)	1.29 ± 0.08	1.28 ± 0.11	1.58 ± 0.04 *^†^	1.23 ± 0.10 ^‡^
UPCR	0.86 ± 0.08	0.83 ± 0.04	2.49 ± 0.41 *^†^	0.98 ± 0.09 ^‡^
U_Prot_V (mg/24 h/g kidney wt)	3.78 ± 0.38	3.35 ± 0.35	8.00 ± 0.74 *^†^	3.55 ± 0.62 ^‡^
AST (U/L)	80.33 ± 4.13	81.50 ± 4.15	114.83 ± 3.9 *^†^	91.50 ± 1.86 *^‡^
ALT (U/L)	24.60 ± 1.33	25.00 ± 1.64	33.20 ± 0.80 *^†^	29.60 ± 0.24 *^‡^

Values are means ± SEM (*n* = 6). Veh: vehicle-treated group; B5 and B50: bisphenol A-treated group at 5 and 50 mg/kg, respectively; B50 + N: BPA (50 mg/kg) plus *N*-acetylcysteine (100 mg/kg) treated group; BUN: blood urea nitrogen; SCr: serum creatinine; CCr: creatinine clearance; UPCR: urine protein-to-creatinine ratio; U_Prot_V: urine protein excretion; AST: aspartate aminotransferase; ALT: alanine aminotransferase. * *p* < 0.05 vs. Veh, ^†^ *p* < 0.05 vs. B5, ^‡^ *p* < 0.05 vs. B50.

## Abbreviations

Ac-SOD2	Acetylated superoxide dismutase 2
AMPK	AMP-activated protein kinase
BPA	Bisphenol A
DCFDA	Dichlorofluorescin diacetate
DRP1	Dynamin-related protein 1
GSH	Reduced glutathione
JC-1	5,5′,6,6′-tetrachloro-1,1′,3,3′-tetraethylbenzimi-dazocarbocyanine iodide
MDA	Malondialdehyde
MFN2	Mitofusin 2
MnSOD	Manganese superoxide dismutase
NAC	*N*-acetylcysteine
NO	Nitric oxide
NOAEL	No observed adverse effect level
p-AMPK	Phosphorylated AMP-activated protein kinase
p-DRP1	Phosphorylated dynamin-related protein 1
PGC-1α	Peroxisome proliferator-activated gamma receptor coactivator-1α
SIRT3	Silent information regulator T3 or sirtuin 3
SOD2	Superoxide dismutase 2
TBST	Tris-buffered saline-Tween 20
UPCR	Urine protein-to-creatinine ratio
VDAC	Voltage-dependent anion channel
